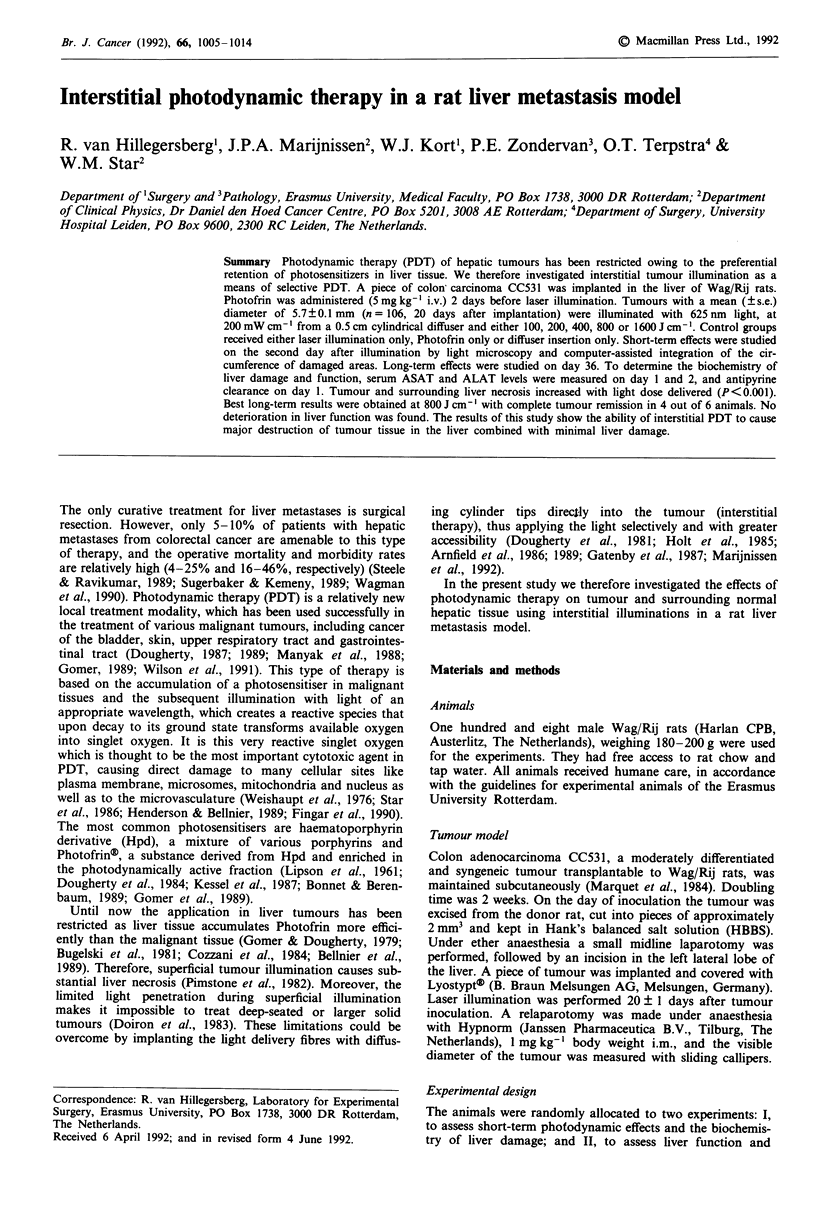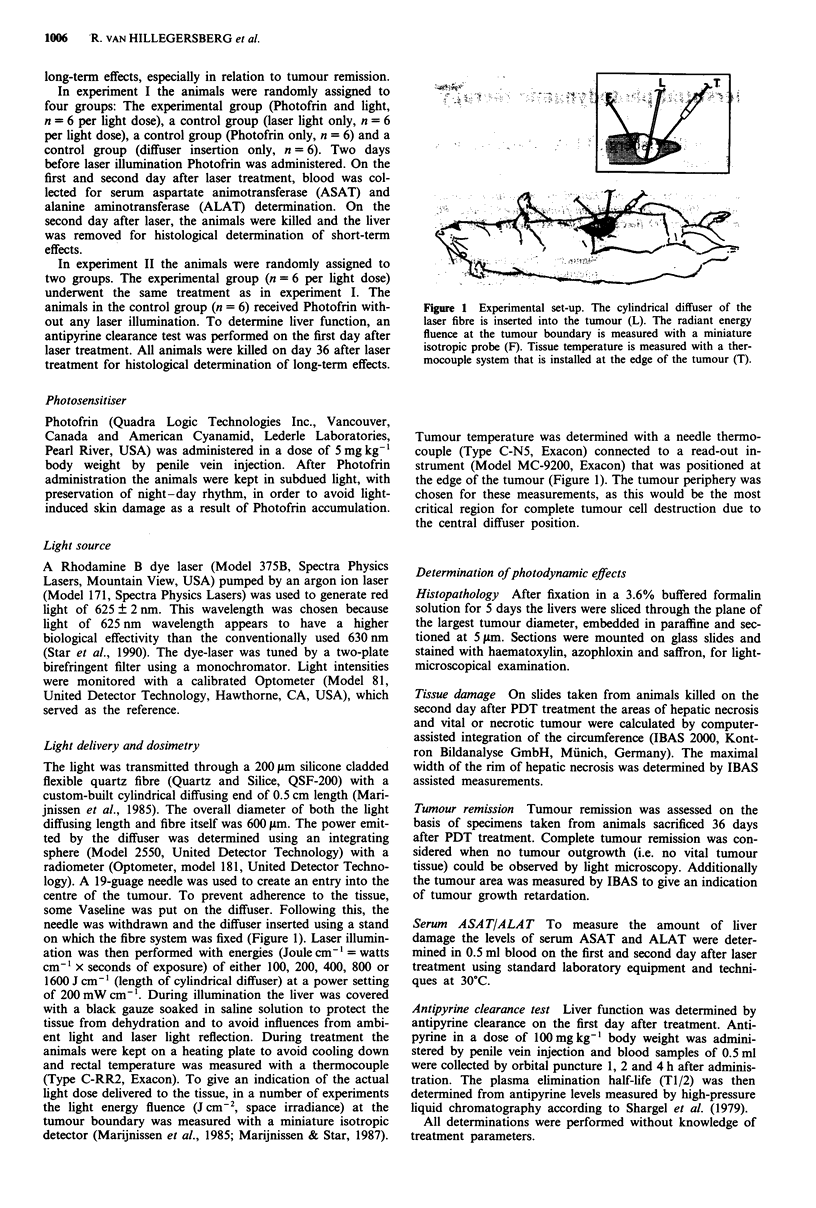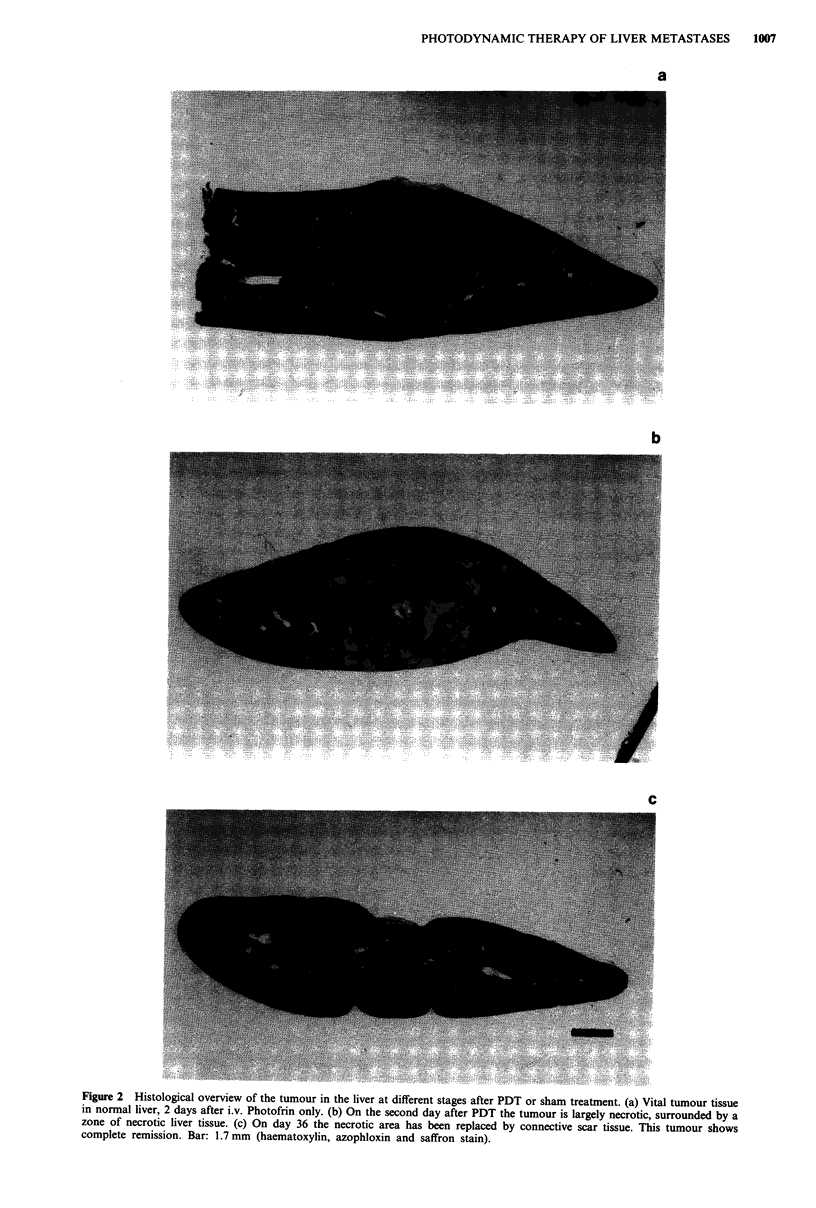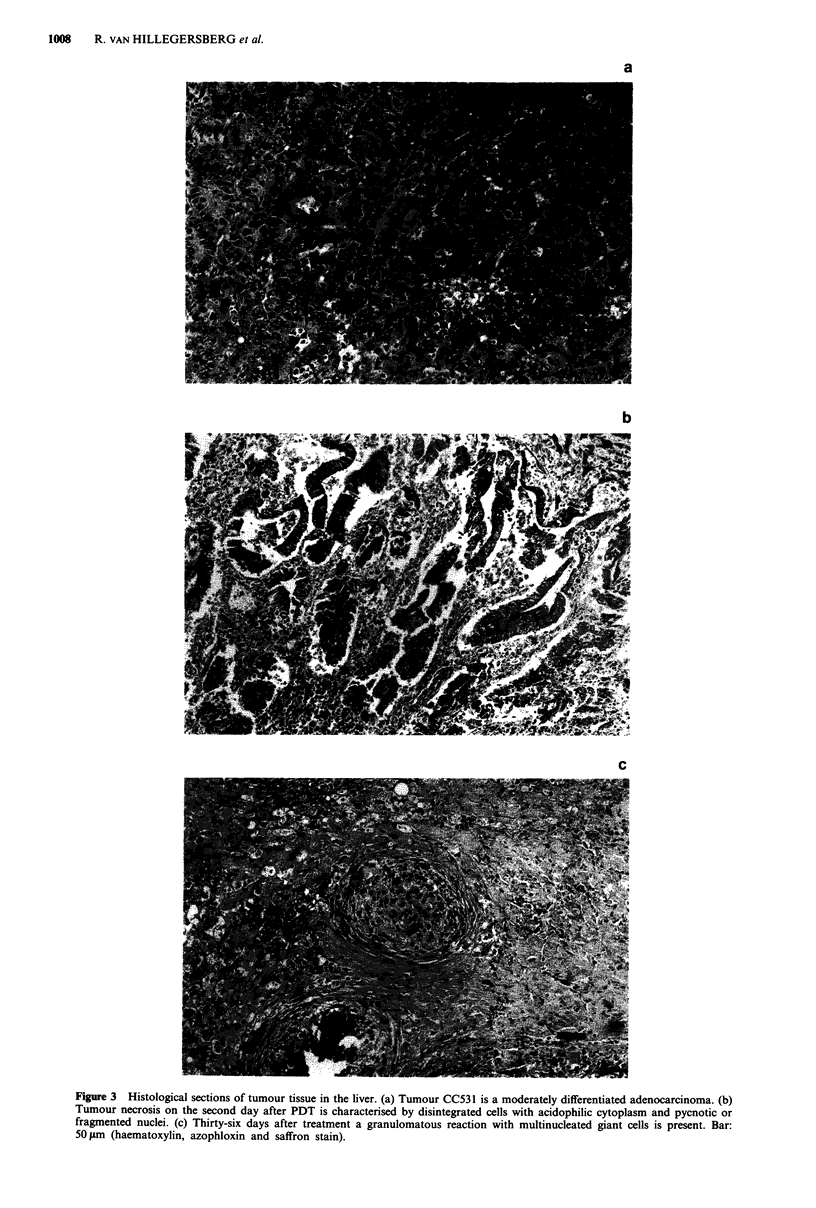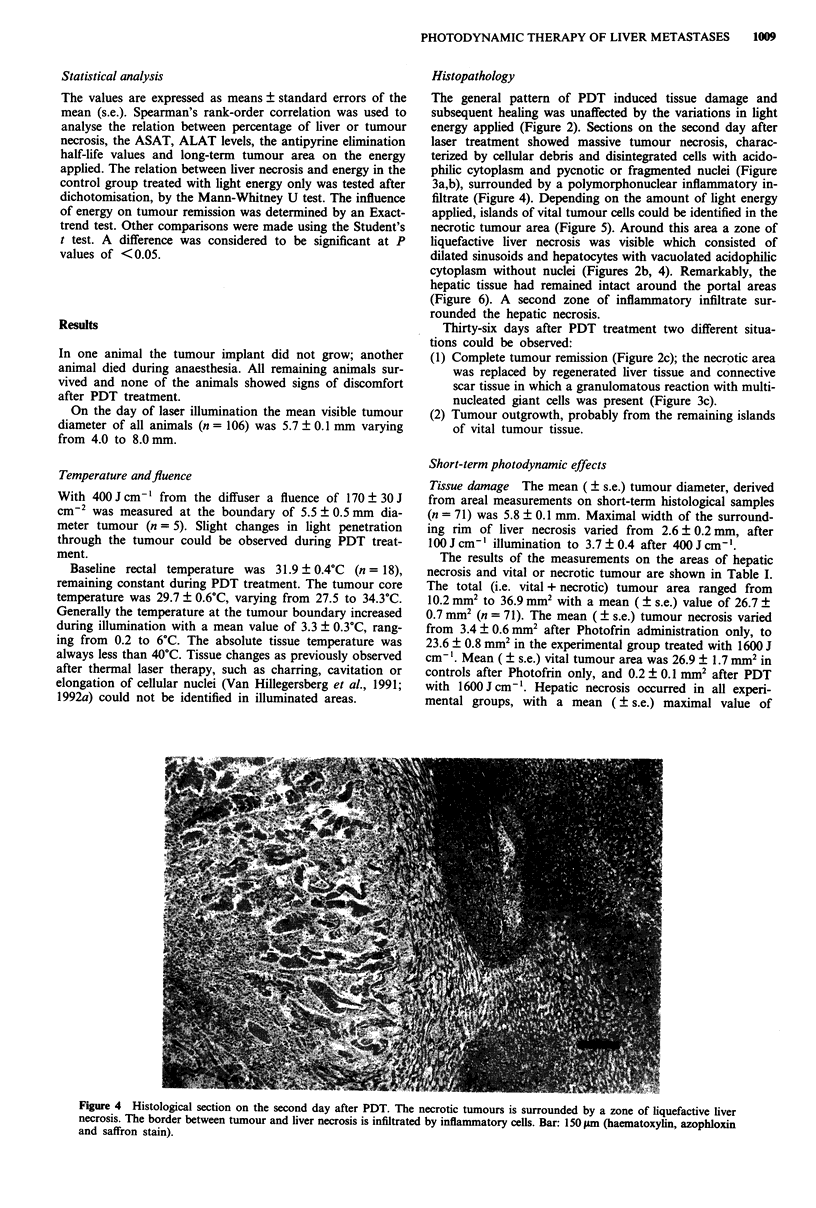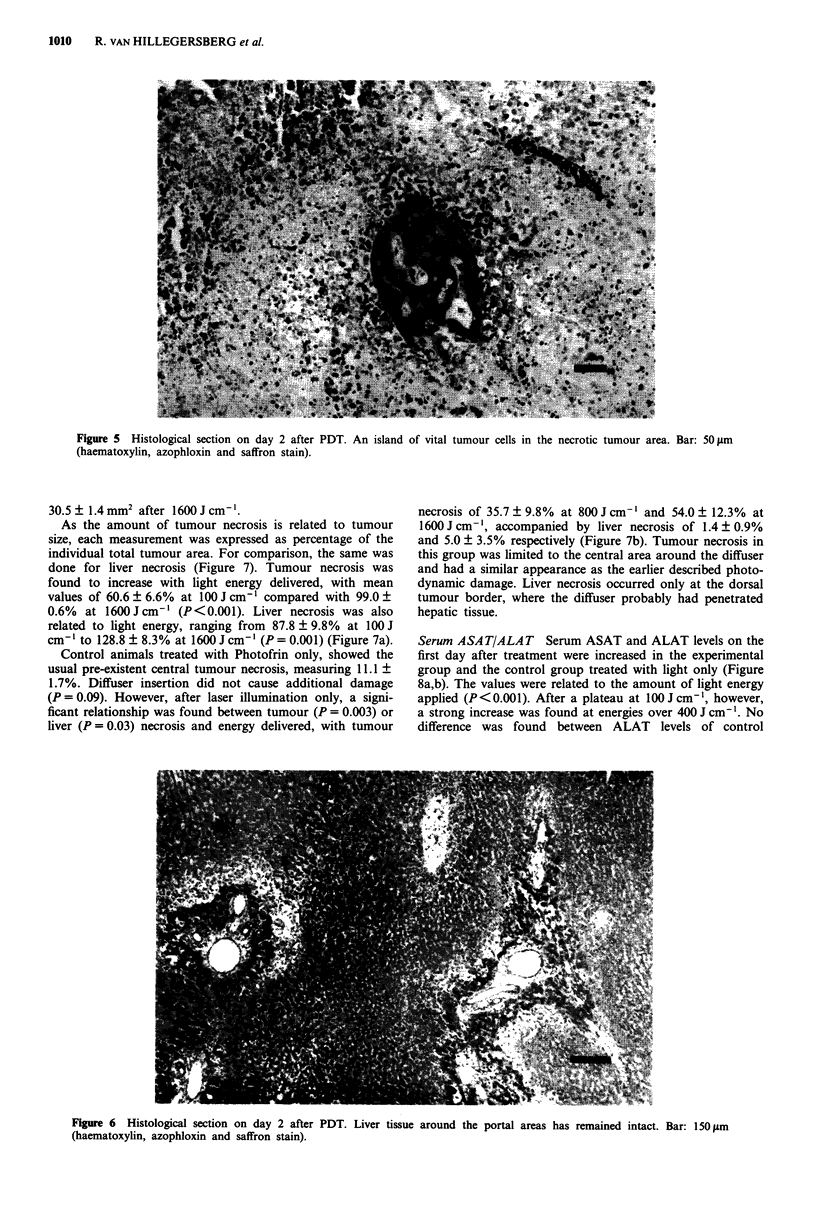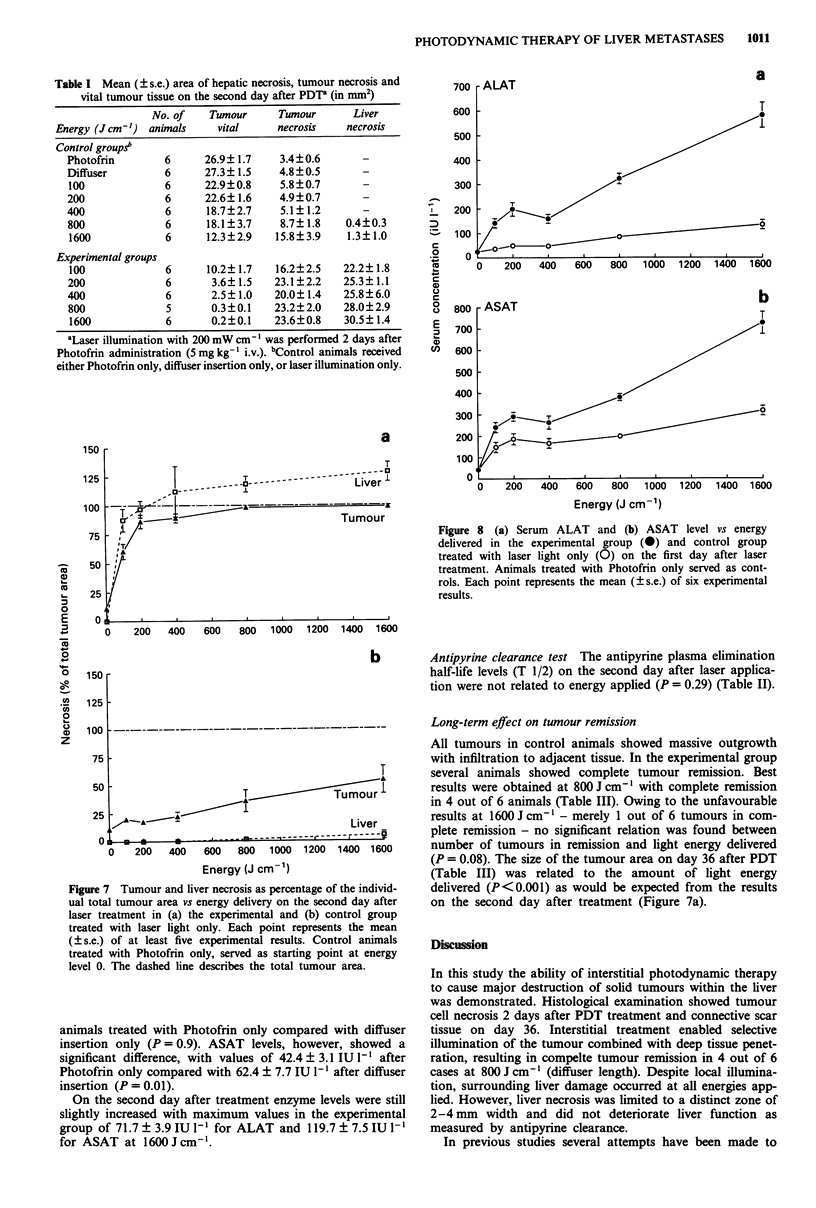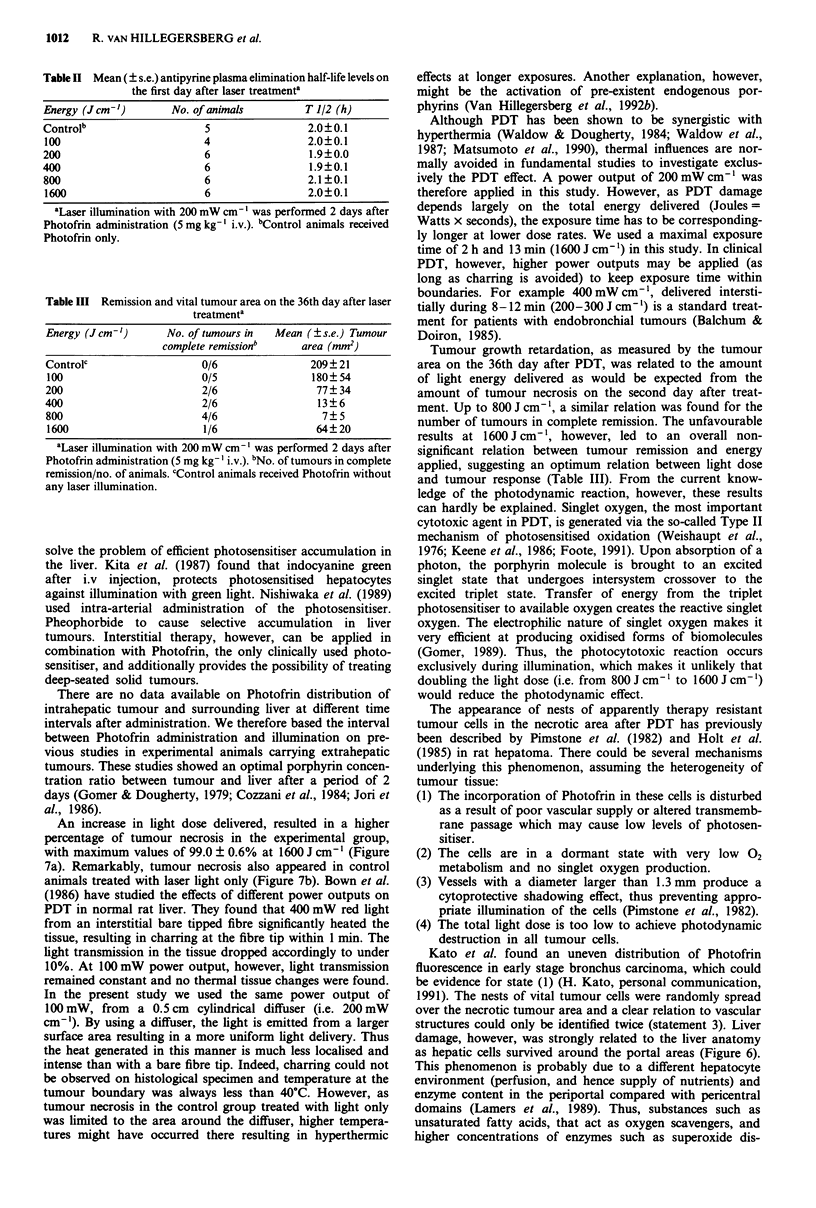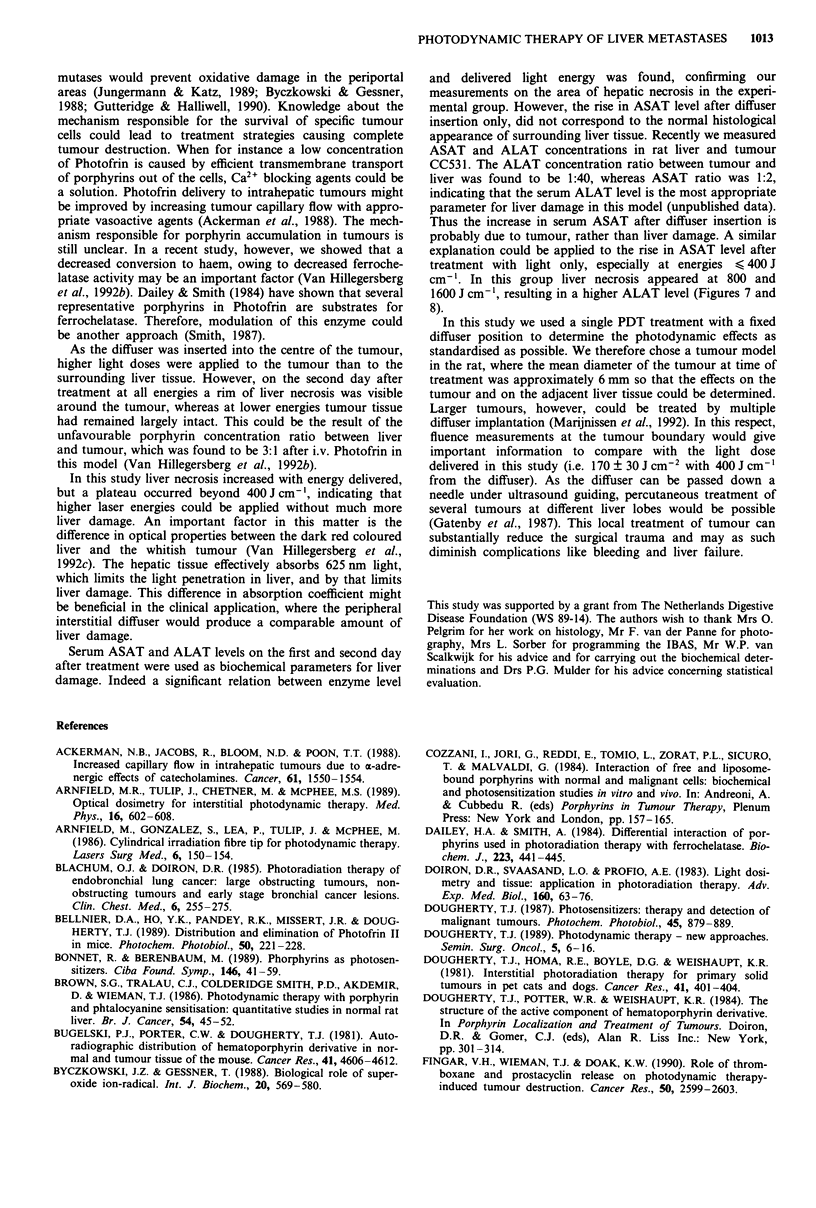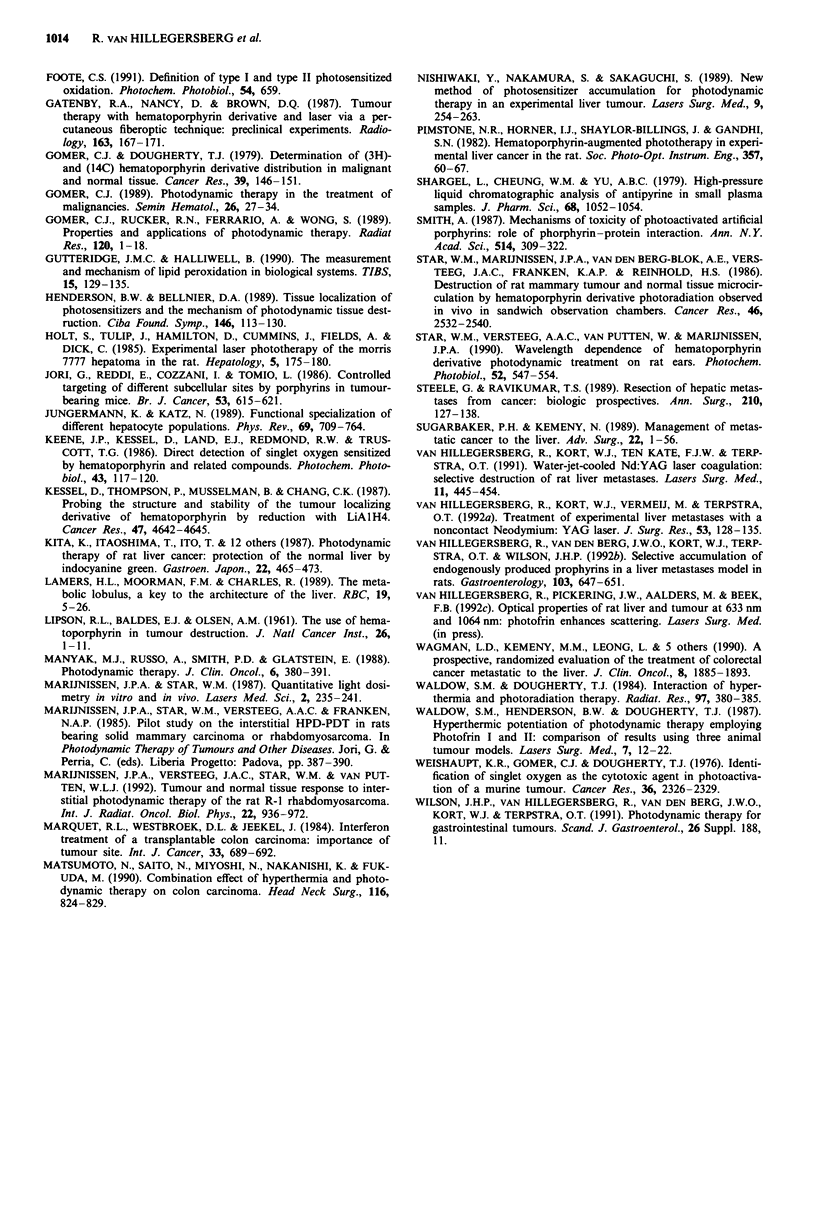# Interstitial photodynamic therapy in a rat liver metastasis model.

**DOI:** 10.1038/bjc.1992.402

**Published:** 1992-12

**Authors:** R. van Hillegersberg, J. P. Marijnissen, W. J. Kort, P. E. Zondervan, O. T. Terpstra, W. M. Star

**Affiliations:** Department of Surgery, Erasmus University, Medical Faculty, Rotterdam, The Netherlands.

## Abstract

**Images:**


Br. J. Cancer (1992), 66, 1005-1014 © Macmillan Press Ltd., 1992

### Interstitial photodynamic therapy in a rat liver metastasis model

R. van Hillegersberg[1], J.P.A. Marijnissen[2], W.J. Kort[1], P.E. Zondervan[3], O.T. Terpstra[4] & W.M. Star[2]

Department of [1]Surgery and [3]Pathology, Erasmus University, Medical Faculty, PO Box 1738, 3000 DR Rotterdam; [2]Department of Clinical Physics, Dr Daniel den Hoed Cancer Centre, PO Box 5201, 3008 AE Rotterdam; [4]Department of Surgery, University Hospital Leiden, PO Box 9600, 2300 RC Leiden, The Netherlands.

**Summary** Photodynamic therapy (PDT) of hepatic tumours has been restricted owing to the preferential retention of photosensitizers in liver tissue. We therefore investigated interstitial tumour illumination as a means of selective PDT. A piece of colon carcinoma CC531 was implanted in the liver of Wag/Rij rats. Photofrin was administered (5 mg kg$^{-1}$ i.v.) 2 days before laser illumination. Tumours with a mean (±s.e.) diameter of 5.7±0.1 mm ($n = 106$, 20 days after implantation) were illuminated with 625 nm light, at 200 mW cm$^{-1}$ from a 0.5 cm cylindrical diffuser and either 100, 200, 400, 800 or 1600 J cm$^{-1}$. Control groups received either laser illumination only, Photofrin only or diffuser insertion only. Short-term effects were studied on the second day after illumination by light microscopy and computer-assisted integration of the circumference of damaged areas. Long-term effects were studied on day 36. To determine the biochemistry of liver damage and function, serum ASAT and ALAT levels were measured on day 1 and 2, and antipyrine clearance on day 1. Tumour and surrounding liver necrosis increased with light dose delivered ($P < 0.001$). Best long-term results were obtained at 800 J cm$^{-1}$ with complete tumour remission in 4 out of 6 animals. No deterioration in liver function was found. The results of this study show the ability of interstitial PDT to cause major destruction of tumour tissue in the liver combined with minimal liver damage.

The only curative treatment for liver metastases is surgical resection. However, only 5-10% of patients with hepatic metastases from colorectal cancer are amenable to this type of therapy, and the operative mortality and morbidity rates are relatively high (4-25% and 16-46%, respectively) (Steele & Ravikumar, 1989; Sugerbaker & Kemeny, 1989; Wagman *et al.*, 1990). Photodynamic therapy (PDT) is a relatively new local treatment modality, which has been used successfully in the treatment of various malignant tumours, including cancer of the bladder, skin, upper respiratory tract and gastrointestinal tract (Dougherty, 1987; 1989; Manyak *et al.*, 1988; Gomer, 1989; Wilson *et al.*, 1991). This type of therapy is based on the accumulation of a photosensitiser in malignant tissues and the subsequent illumination with light of an appropriate wavelength, which creates a reactive species that upon decay to its ground state transforms available oxygen into singlet oxygen. It is this very reactive singlet oxygen which is thought to be the most important cytotoxic agent in PDT, causing direct damage to many cellular sites like plasma membrane, microsomes, mitochondria and nucleus as well as to the microvasculature (Weishaupt *et al.*, 1976; Star *et al.*, 1986; Henderson & Bellnier, 1989; Fingar *et al.*, 1990). The most common photosensitisers are haematoporphyrin derivative (Hpd), a mixture of various porphyrins and Photofrin®, a substance derived from Hpd and enriched in the photodynamically active fraction (Lipson *et al.*, 1961; Dougherty *et al.*, 1984; Kessel *et al.*, 1987; Bonnet & Berenbaum, 1989; Gomer *et al.*, 1989).

Until now the application in liver tumours has been restricted as liver tissue accumulates Photofrin more efficiently than the malignant tissue (Gomer & Dougherty, 1979; Bugelski *et al.*, 1981; Cozzani *et al.*, 1984; Bellnier *et al.*, 1989). Therefore, superficial tumour illumination causes substantial liver necrosis (Pimstone *et al.*, 1982). Moreover, the limited light penetration during superficial illumination makes it impossible to treat deep-seated or larger solid tumours (Doiron *et al.*, 1983). These limitations could be overcome by implanting the light delivery fibres with diffusing cylinder tips directly into the tumour (interstitial therapy), thus applying the light selectively and with greater accessibility (Dougherty *et al.*, 1981; Holt *et al.*, 1985; Arnfield *et al.*, 1986; 1989; Gatenby *et al.*, 1987; Marijnissen *et al.*, 1992).

In the present study we therefore investigated the effects of photodynamic therapy on tumour and surrounding normal hepatic tissue using interstitial illuminations in a rat liver metastasis model.

### Materials and methods

### Animals

One hundred and eight male Wag/Rij rats (Harlan CPB, Austerlitz, The Netherlands), weighing 180-200 g were used for the experiments. They had free access to rat chow and tap water. All animals received humane care, in accordance with the guidelines for experimental animals of the Erasmus University Rotterdam.

### Tumour model

Colon adenocarcinoma CC531, a moderately differentiated and syngeneic tumour transplantable to Wag/Rij rats, was maintained subcutaneously (Marquet *et al.*, 1984). Doubling time was 2 weeks. On the day of inoculation the tumour was excised from the donor rat, cut into pieces of approximately 2 mm$^3$ and kept in Hank's balanced salt solution (HBBS). Under ether anaesthesia a small midline laparotomy was performed, followed by an incision in the left lateral lobe of the liver. A piece of tumour was implanted and covered with Lyostypt® (B. Braun Melsungen AG, Melsungen, Germany). Laser illumination was performed 20 ± 1 days after tumour inoculation. A relaparotomy was made under anaesthesia with Hypnorm (Janssen Pharmaceutica B.V., Tilburg, The Netherlands), 1 mg kg$^{-1}$ body weight i.m., and the visible diameter of the tumour was measured with sliding callipers.

### Experimental design

The animals were randomly allocated to two experiments: I, to assess short-term photodynamic effects and the biochemistry of liver damage; and II, to assess liver function and

Correspondence: R. van Hillegersberg, Laboratory for Experimental Surgery, Erasmus University, PO Box 1738, 3000 DR Rotterdam, The Netherlands.
Received 6 April 1992; and in revised form 4 June 1992.

long-term effects, especially in relation to tumour remission.

In experiment I the animals were randomly assigned to four groups: The experimental group (Photofrin and light, $n = 6$ per light dose), a control group (laser light only, $n = 6$ per light dose), a control group (Photofrin only, $n = 6$) and a control group (diffuser insertion only, $n = 6$). Two days before laser illumination Photofrin was administered. On the first and second day after laser treatment, blood was collected for serum aspartate animotransferase (ASAT) and alanine aminotransferase (ALAT) determination. On the second day after laser, the animals were killed and the liver was removed for histological determination of short-term effects.

In experiment II the animals were randomly assigned to two groups. The experimental group ($n = 6$ per light dose) underwent the same treatment as in experiment I. The animals in the control group ($n = 6$) received Photofrin without any laser illumination. To determine liver function, an antipyrine clearance test was performed on the first day after laser treatment. All animals were killed on day 36 after laser treatment for histological determination of long-term effects.

**Figure 1** Experimental set-up. The cylindrical diffuser of the laser fibre is inserted into the tumour (L). The radiant energy fluence at the tumour boundary is measured with a miniature isotropic probe (F). Tissue temperature is measured with a thermocouple system that is installed at the edge of the tumour (T).

*Photosensitiser*

Photofrin (Quadra Logic Technologies Inc., Vancouver, Canada and American Cyanamid, Lederle Laboratories, Pearl River, USA) was administered in a dose of 5 mg $kg^{-1}$ body weight by penile vein injection. After Photofrin administration the animals were kept in subdued light, with preservation of night–day rhythm, in order to avoid light-induced skin damage as a result of Photofrin accumulation.

*Light source*

A Rhodamine B dye laser (Model 375B, Spectra Physics Lasers, Mountain View, USA) pumped by an argon ion laser (Model 171, Spectra Physics Lasers) was used to generate red light of $625 \pm 2$ nm. This wavelength was chosen because light of 625 nm wavelength appears to have a higher biological effectivity than the conventionally used 630 nm (Star *et al.*, 1990). The dye-laser was tuned by a two-plate birefringent filter using a monochromator. Light intensities were monitored with a calibrated Optometer (Model 81, United Detector Technology, Hawthorne, CA, USA), which served as the reference.

*Light delivery and dosimetry*

The light was transmitted through a 200 μm silicone cladded flexible quartz fibre (Quartz and Silice, QSF-200) with a custom-built cylindrical diffusing end of 0.5 cm length (Marijnissen *et al.*, 1985). The overall diameter of both the light diffusing length and fibre itself was 600 μm. The power emitted by the diffuser was determined using an integrating sphere (Model 2550, United Detector Technology) with a radiometer (Optometer, model 181, United Detector Technology). A 19-guage needle was used to create an entry into the centre of the tumour. To prevent adherence to the tissue, some Vaseline was put on the diffuser. Following this, the needle was withdrawn and the diffuser inserted using a stand on which the fibre system was fixed (Figure 1). Laser illumination was then performed with energies (Joule $cm^{-1}$ = watts $cm^{-1}$ × seconds of exposure) of either 100, 200, 400, 800 or 1600 J $cm^{-1}$ (length of cylindrical diffuser) at a power setting of 200 mW $cm^{-1}$. During illumination the liver was covered with a black gauze soaked in saline solution to protect the tissue from dehydration and to avoid influences from ambient light and laser light reflection. During treatment the animals were kept on a heating plate to avoid cooling down and rectal temperature was measured with a thermocouple (Type C-RR2, Exacon). To give an indication of the actual light dose delivered to the tissue, in a number of experiments the light energy fluence (J $cm^{-2}$, space irradiance) at the tumour boundary was measured with a miniature isotropic detector (Marijnissen *et al.*, 1985; Marijnissen & Star, 1987).

Tumour temperature was determined with a needle thermocouple (Type C-N5, Exacon) connected to a read-out instrument (Model MC-9200, Exacon) that was positioned at the edge of the tumour (Figure 1). The tumour periphery was chosen for these measurements, as this would be the most critical region for complete tumour cell destruction due to the central diffuser position.

*Determination of photodynamic effects*

*Histopathology* After fixation in a 3.6% buffered formalin solution for 5 days the livers were sliced through the plane of the largest tumour diameter, embedded in paraffine and sectioned at 5 μm. Sections were mounted on glass slides and stained with haematoxylin, azophloxin and saffron, for light-microscopical examination.

*Tissue damage* On slides taken from animals killed on the second day after PDT treatment the areas of hepatic necrosis and vital or necrotic tumour were calculated by computer-assisted integration of the circumference (IBAS 2000, Kontron Bildanalyse GmbH, Münich, Germany). The maximal width of the rim of hepatic necrosis was determined by IBAS assisted measurements.

*Tumour remission* Tumour remission was assessed on the basis of specimens taken from animals sacrificed 36 days after PDT treatment. Complete tumour remission was considered when no tumour outgrowth (i.e. no vital tumour tissue) could be observed by light microscopy. Additionally the tumour area was measured by IBAS to give an indication of tumour growth retardation.

*Serum ASAT/ALAT* To measure the amount of liver damage the levels of serum ASAT and ALAT were determined in 0.5 ml blood on the first and second day after laser treatment using standard laboratory equipment and techniques at 30°C.

*Antipyrine clearance test* Liver function was determined by antipyrine clearance on the first day after treatment. Antipyrine in a dose of 100 mg $kg^{-1}$ body weight was administered by penile vein injection and blood samples of 0.5 ml were collected by orbital puncture 1, 2 and 4 h after administration. The plasma elimination half-life (T1/2) was then determined from antipyrine levels measured by high-pressure liquid chromatography according to Shargel *et al.* (1979).

All determinations were performed without knowledge of treatment parameters.

**Figure 2** Histological overview of the tumour in the liver at different stages after PDT or sham treatment. (a) Vital tumour tissue in normal liver, 2 days after i.v. Photofrin only. (b) On the second day after PDT the tumour is largely necrotic, surrounded by a zone of necrotic liver tissue. (c) On day 36 the necrotic area has been replaced by connective scar tissue. This tumour shows complete remission. Bar: 1.7 mm (haematoxylin, azophloxin and saffron stain).

**Figure 3** Histological sections of tumour tissue in the liver. (a) Tumour CC531 is a moderately differentiated adenocarcinoma. (b) Tumour necrosis on the second day after PDT is characterised by disintegrated cells with acidophilic cytoplasm and pycnotic or fragmented nuclei. (c) Thirty-six days after treatment a granulomatous reaction with multinucleated giant cells is present. Bar: 50 μm (haematoxylin, azophloxin and saffron stain).

*Statistical analysis*

The values are expressed as means ± standard errors of the mean (s.e.). Spearman's rank-order correlation was used to analyse the relation between percentage of liver or tumour necrosis, the ASAT, ALAT levels, the antipyrine elimination half-life values and long-term tumour area on the energy applied. The relation between liver necrosis and energy in the control group treated with light energy only was tested after dichotomisation, by the Mann-Whitney U test. The influence of energy on tumour remission was determined by an Exact-trend test. Other comparisons were made using the Student's *t* test. A difference was considered to be significant at *P* values of $<0.05$.

### Results

In one animal the tumour implant did not grow; another animal died during anaesthesia. All remaining animals survived and none of the animals showed signs of discomfort after PDT treatment.

On the day of laser illumination the mean visible tumour diameter of all animals ($n = 106$) was 5.7 ± 0.1 mm varying from 4.0 to 8.0 mm.

*Temperature and fluence*

With 400 J $cm^{-1}$ from the diffuser a fluence of 170 ± 30 J $cm^{-2}$ was measured at the boundary of 5.5 ± 0.5 mm diameter tumour ($n = 5$). Slight changes in light penetration through the tumour could be observed during PDT treatment.

Baseline rectal temperature was 31.9 ± 0.4°C ($n = 18$), remaining constant during PDT treatment. The tumour core temperature was 29.7 ± 0.6°C, varying from 27.5 to 34.3°C. Generally the temperature at the tumour boundary increased during illumination with a mean value of 3.3 ± 0.3°C, ranging from 0.2 to 6°C. The absolute tissue temperature was always less than 40°C. Tissue changes as previously observed after thermal laser therapy, such as charring, cavitation or elongation of cellular nuclei (Van Hillegersberg *et al.*, 1991; 1992*a*) could not be identified in illuminated areas.

*Histopathology*

The general pattern of PDT induced tissue damage and subsequent healing was unaffected by the variations in light energy applied (Figure 2). Sections on the second day after laser treatment showed massive tumour necrosis, characterized by cellular debris and disintegrated cells with acidophilic cytoplasm and pycnotic or fragmented nuclei (Figure 3a,b), surrounded by a polymorphonuclear inflammatory infiltrate (Figure 4). Depending on the amount of light energy applied, islands of vital tumour cells could be identified in the necrotic tumour area (Figure 5). Around this area a zone of liquefactive liver necrosis was visible which consisted of dilated sinusoids and hepatocytes with vacuolated acidophilic cytoplasm without nuclei (Figures 2b, 4). Remarkably, the hepatic tissue had remained intact around the portal areas (Figure 6). A second zone of inflammatory infiltrate surrounded the hepatic necrosis.

Thirty-six days after PDT treatment two different situations could be observed:

(1) Complete tumour remission (Figure 2c); the necrotic area was replaced by regenerated liver tissue and connective scar tissue in which a granulomatous reaction with multinucleated giant cells was present (Figure 3c).

(2) Tumour outgrowth, probably from the remaining islands of vital tumour tissue.

*Short-term photodynamic effects*

*Tissue damage* The mean (± s.e.) tumour diameter, derived from areal measurements on short-term histological samples ($n = 71$) was 5.8 ± 0.1 mm. Maximal width of the surrounding rim of liver necrosis varied from 2.6 ± 0.2 mm, after 100 J $cm^{-1}$ illumination to 3.7 ± 0.4 after 400 J $cm^{-1}$.

The results of the measurements on the areas of hepatic necrosis and vital or necrotic tumour are shown in Table I. The total (i.e. vital + necrotic) tumour area ranged from 10.2 $mm^2$ to 36.9 $mm^2$ with a mean (± s.e.) value of 26.7 ± 0.7 $mm^2$ ($n = 71$). The mean (± s.e.) tumour necrosis varied from 3.4 ± 0.6 $mm^2$ after Photofrin administration only, to 23.6 ± 0.8 $mm^2$ in the experimental group treated with 1600 J $cm^{-1}$. Mean (± s.e.) vital tumour area was 26.9 ± 1.7 $mm^2$ in controls after Photofrin only, and 0.2 ± 0.1 $mm^2$ after PDT with 1600 J $cm^{-1}$. Hepatic necrosis occurred in all experimental groups, with a mean (± s.e.) maximal value of

**Figure 4** Histological section on the second day after PDT. The necrotic tumours is surrounded by a zone of liquefactive liver necrosis. The border between tumour and liver necrosis is infiltrated by inflammatory cells. Bar: 150 μm (haematoxylin, azophloxin and saffron stain).

**Figure 5** Histological section on day 2 after PDT. An island of vital tumour cells in the necrotic tumour area. Bar: 50 μm (haematoxylin, azophloxin and saffron stain).

30.5 ± 1.4 mm$^2$ after 1600 J cm$^{-1}$.

As the amount of tumour necrosis is related to tumour size, each measurement was expressed as percentage of the individual total tumour area. For comparison, the same was done for liver necrosis (Figure 7). Tumour necrosis was found to increase with light energy delivered, with mean values of 60.6 ± 6.6% at 100 J cm$^{-1}$ compared with 99.0 ± 0.6% at 1600 J cm$^{-1}$ ($P < 0.001$). Liver necrosis was also related to light energy, ranging from 87.8 ± 9.8% at 100 J cm$^{-1}$ to 128.8 ± 8.3% at 1600 J cm$^{-1}$ ($P = 0.001$) (Figure 7a).

Control animals treated with Photofrin only, showed the usual pre-existent central tumour necrosis, measuring 11.1 ± 1.7%. Diffuser insertion did not cause additional damage ($P = 0.09$). However, after laser illumination only, a significant relationship was found between tumour ($P = 0.003$) or liver ($P = 0.03$) necrosis and energy delivered, with tumour necrosis of 35.7 ± 9.8% at 800 J cm$^{-1}$ and 54.0 ± 12.3% at 1600 J cm$^{-1}$, accompanied by liver necrosis of 1.4 ± 0.9% and 5.0 ± 3.5% respectively (Figure 7b). Tumour necrosis in this group was limited to the central area around the diffuser and had a similar appearance as the earlier described photodynamic damage. Liver necrosis occurred only at the dorsal tumour border, where the diffuser probably had penetrated hepatic tissue.

*Serum ASAT/ALAT* Serum ASAT and ALAT levels on the first day after treatment were increased in the experimental group and the control group treated with light only (Figure 8a,b). The values were related to the amount of light energy applied ($P < 0.001$). After a plateau at 100 J cm$^{-1}$, however, a strong increase was found at energies over 400 J cm$^{-1}$. No difference was found between ALAT levels of control

**Figure 6** Histological section on day 2 after PDT. Liver tissue around the portal areas has remained intact. Bar: 150 μm (haematoxylin, azophloxin and saffron stain).

**Table I** Mean (±s.e.) area of hepatic necrosis, tumour necrosis and vital tumour tissue on the second day after PDT[a] (in mm²)

| *Energy (J cm⁻¹)* | *No. of animals* | *Tumour vital* | *Tumour necrosis* | *Liver necrosis* |
|---|---|---|---|---|
| *Control groups*[b] | | | | |
| Photofrin | 6 | 26.9±1.7 | 3.4±0.6 | – |
| Diffuser | 6 | 27.3±1.5 | 4.8±0.5 | – |
| 100 | 6 | 22.9±0.8 | 5.8±0.7 | – |
| 200 | 6 | 22.6±1.6 | 4.9±0.7 | – |
| 400 | 6 | 18.7±2.7 | 5.1±1.2 | – |
| 800 | 6 | 18.1±3.7 | 8.7±1.8 | 0.4±0.3 |
| 1600 | 6 | 12.3±2.9 | 15.8±3.9 | 1.3±1.0 |
| *Experimental groups* | | | | |
| 100 | 6 | 10.2±1.7 | 16.2±2.5 | 22.2±1.8 |
| 200 | 6 | 3.6±1.5 | 23.1±2.2 | 25.3±1.1 |
| 400 | 6 | 2.5±1.0 | 20.0±1.4 | 25.8±6.0 |
| 800 | 5 | 0.3±0.1 | 23.2±2.0 | 28.0±2.9 |
| 1600 | 6 | 0.2±0.1 | 23.6±0.8 | 30.5±1.4 |

[a]Laser illumination with 200 mW $cm^{-1}$ was performed 2 days after Photofrin administration (5 mg $kg^{-1}$ i.v.). [b]Control animals received either Photofrin only, diffuser insertion only, or laser illumination only.

**Figure 7** Tumour and liver necrosis as percentage of the individual total tumour area *vs* energy delivery on the second day after laser treatment in (a) the experimental and (b) control group treated with laser light only. Each point represents the mean (±s.e.) of at least five experimental results. Control animals treated with Photofrin only, served as starting point at energy level 0. The dashed line describes the total tumour area.

animals treated with Photofrin only compared with diffuser insertion only ($P = 0.9$). ASAT levels, however, showed a significant difference, with values of 42.4 ± 3.1 IU $l^{-1}$ after Photofrin only compared with 62.4 ± 7.7 IU $l^{-1}$ after diffuser insertion ($P = 0.01$).

On the second day after treatment enzyme levels were still slightly increased with maximum values in the experimental group of 71.7 ± 3.9 IU $l^{-1}$ for ALAT and 119.7 ± 7.5 IU $l^{-1}$ for ASAT at 1600 J $cm^{-1}$.

**Figure 8** (**a**) Serum ALAT and (**b**) ASAT level *vs* energy delivered in the experimental group (●) and control group treated with laser light only (○) on the first day after laser treatment. Animals treated with Photofrin only served as controls. Each point represents the mean (±s.e.) of six experimental results.

*Antipyrine clearance test* The antipyrine plasma elimination half-life levels (T 1/2) on the second day after laser application were not related to energy applied ($P = 0.29$) (Table II).

### *Long-term effect on tumour remission*

All tumours in control animals showed massive outgrowth with infiltration to adjacent tissue. In the experimental group several animals showed complete tumour remission. Best results were obtained at 800 J $cm^{-1}$ with complete remission in 4 out of 6 animals (Table III). Owing to the unfavourable results at 1600 J $cm^{-1}$ – merely 1 out of 6 tumours in complete remission – no significant relation was found between number of tumours in remission and light energy delivered ($P = 0.08$). The size of the tumour area on day 36 after PDT (Table III) was related to the amount of light energy delivered ($P < 0.001$) as would be expected from the results on the second day after treatment (Figure 7a).

### Discussion

In this study the ability of interstitial photodynamic therapy to cause major destruction of solid tumours within the liver was demonstrated. Histological examination showed tumour cell necrosis 2 days after PDT treatment and connective scar tissue on day 36. Interstitial treatment enabled selective illumination of the tumour combined with deep tissue penetration, resulting in compelte tumour remission in 4 out of 6 cases at 800 J $cm^{-1}$ (diffuser length). Despite local illumination, surrounding liver damage occurred at all energies applied. However, liver necrosis was limited to a distinct zone of 2–4 mm width and did not deteriorate liver function as measured by antipyrine clearance.

In previous studies several attempts have been made to

**Table II** Mean (±s.e.) antipyrine plasma elimination half-life levels on the first day after laser treatment[a]

| Energy ($J\ cm^{-1}$) | No. of animals | T 1/2 (h) |
|---|---|---|
| Control[b] | 5 | 2.0±0.1 |
| 100 | 4 | 2.0±0.1 |
| 200 | 6 | 1.9±0.0 |
| 400 | 6 | 1.9±0.1 |
| 800 | 6 | 2.1±0.1 |
| 1600 | 6 | 2.0±0.1 |

[a]Laser illumination with 200 mW $cm^{-1}$ was performed 2 days after Photofrin administration (5 mg $kg^{-1}$ i.v.). [b]Control animals received Photofrin only.

**Table III** Remission and vital tumour area on the 36th day after laser treatment[a]

| Energy ($J\ cm^{-1}$) | No. of tumours in complete remission[b] | Mean (±s.e.) Tumour area ($mm^2$) |
|---|---|---|
| Control[c] | 0/6 | 209±21 |
| 100 | 0/5 | 180±54 |
| 200 | 2/6 | 77±34 |
| 400 | 2/6 | 13±6 |
| 800 | 4/6 | 7±5 |
| 1600 | 1/6 | 64±20 |

[a]Laser illumination with 200 mW $cm^{-1}$ was performed 2 days after Photofrin administration (5 mg $kg^{-1}$ i.v.). [b]No. of tumours in complete remission/no. of animals. [c]Control animals received Photofrin without any laser illumination.

solve the problem of efficient photosensitiser accumulation in the liver. Kita *et al.* (1987) found that indocyanine green after i.v injection, protects photosensitised hepatocytes against illumination with green light. Nishiwaka *et al.* (1989) used intra-arterial administration of the photosensitiser. Pheophorbide to cause selective accumulation in liver tumours. Interstitial therapy, however, can be applied in combination with Photofrin, the only clinically used photosensitiser, and additionally provides the possibility of treating deep-seated solid tumours.

There are no data available on Photofrin distribution of intrahepatic tumour and surrounding liver at different time intervals after administration. We therefore based the interval between Photofrin administration and illumination on previous studies in experimental animals carrying extrahepatic tumours. These studies showed an optimal porphyrin concentration ratio between tumour and liver after a period of 2 days (Gomer & Dougherty, 1979; Cozzani *et al.*, 1984; Jori *et al.*, 1986).

An increase in light dose delivered, resulted in a higher percentage of tumour necrosis in the experimental group, with maximum values of 99.0 ± 0.6% at 1600 J $cm^{-1}$ (Figure 7a). Remarkably, tumour necrosis also appeared in control animals treated with laser light only (Figure 7b). Bown *et al.* (1986) have studied the effects of different power outputs on PDT in normal rat liver. They found that 400 mW red light from an interstitial bare tipped fibre significantly heated the tissue, resulting in charring at the fibre tip within 1 min. The light transmission in the tissue dropped accordingly to under 10%. At 100 mW power output, however, light transmission remained constant and no thermal tissue changes were found. In the present study we used the same power output of 100 mW, from a 0.5 cm cylindrical diffuser (i.e. 200 mW $cm^{-1}$). By using a diffuser, the light is emitted from a larger surface area resulting in a more uniform light delivery. Thus the heat generated in this manner is much less localised and intense than with a bare fibre tip. Indeed, charring could not be observed on histological specimen and temperature at the tumour boundary was always less than 40°C. However, as tumour necrosis in the control group treated with light only was limited to the area around the diffuser, higher temperatures might have occurred there resulting in hyperthermic effects at longer exposures. Another explanation, however, might be the activation of pre-existent endogenous porphyrins (Van Hillegersberg *et al.*, 1992*b*).

Although PDT has been shown to be synergistic with hyperthermia (Waldow & Dougherty, 1984; Waldow *et al.*, 1987; Matsumoto *et al.*, 1990), thermal influences are normally avoided in fundamental studies to investigate exclusively the PDT effect. A power output of 200 mW $cm^{-1}$ was therefore applied in this study. However, as PDT damage depends largely on the total energy delivered (Joules = Watts × seconds), the exposure time has to be correspondingly longer at lower dose rates. We used a maximal exposure time of 2 h and 13 min (1600 J $cm^{-1}$) in this study. In clinical PDT, however, higher power outputs may be applied (as long as charring is avoided) to keep exposure time within boundaries. For example 400 mW $cm^{-1}$, delivered interstitially during 8–12 min (200–300 J $cm^{-1}$) is a standard treatment for patients with endobronchial tumours (Balchum & Doiron, 1985).

Tumour growth retardation, as measured by the tumour area on the 36th day after PDT, was related to the amount of light energy delivered as would be expected from the amount of tumour necrosis on the second day after treatment. Up to 800 J $cm^{-1}$, a similar relation was found for the number of tumours in complete remission. The unfavourable results at 1600 J $cm^{-1}$, however, led to an overall non-significant relation between tumour remission and energy applied, suggesting an optimum relation between light dose and tumour response (Table III). From the current knowledge of the photodynamic reaction, however, these results can hardly be explained. Singlet oxygen, the most important cytotoxic agent in PDT, is generated via the so-called Type II mechanism of photosensitised oxidation (Weishaupt *et al.*, 1976; Keene *et al.*, 1986; Foote, 1991). Upon absorption of a photon, the porphyrin molecule is brought to an excited singlet state that undergoes intersystem crossover to the excited triplet state. Transfer of energy from the triplet photosensitiser to available oxygen creates the reactive singlet oxygen. The electrophilic nature of singlet oxygen makes it very efficient at producing oxidised forms of biomolecules (Gomer, 1989). Thus, the photocytotoxic reaction occurs exclusively during illumination, which makes it unlikely that doubling the light dose (i.e. from 800 J $cm^{-1}$ to 1600 J $cm^{-1}$) would reduce the photodynamic effect.

The appearance of nests of apparently therapy resistant tumour cells in the necrotic area after PDT has previously been described by Pimstone *et al.* (1982) and Holt *et al.* (1985) in rat hepatoma. There could be several mechanisms underlying this phenomenon, assuming the heterogeneity of tumour tissue:

(1) The incorporation of Photofrin in these cells is disturbed as a result of poor vascular supply or altered transmembrane passage which may cause low levels of photosensitiser.
(2) The cells are in a dormant state with very low $O_2$ metabolism and no singlet oxygen production.
(3) Vessels with a diameter larger than 1.3 mm produce a cytoprotective shadowing effect, thus preventing appropriate illumination of the cells (Pimstone *et al.*, 1982).
(4) The total light dose is too low to achieve photodynamic destruction in all tumour cells.

Kato *et al.* found an uneven distribution of Photofrin fluorescence in early stage bronchus carcinoma, which could be evidence for state (1) (H. Kato, personal communication, 1991). The nests of vital tumour cells were randomly spread over the necrotic tumour area and a clear relation to vascular structures could only be identified twice (statement 3). Liver damage, however, was strongly related to the liver anatomy as hepatic cells survived around the portal areas (Figure 6). This phenomenon is probably due to a different hepatocyte environment (perfusion, and hence supply of nutrients) and enzyme content in the periportal compared with pericentral domains (Lamers *et al.*, 1989). Thus, substances such as unsaturated fatty acids, that act as oxygen scavengers, and higher concentrations of enzymes such as superoxide dismutases would prevent oxidative damage in the periportal areas (Jungermann & Katz, 1989; Byczkowski & Gessner, 1988; Gutteridge & Halliwell, 1990). Knowledge about the mechanism responsible for the survival of specific tumour cells could lead to treatment strategies causing complete tumour destruction. When for instance a low concentration of Photofrin is caused by efficient transmembrane transport of porphyrins out of the cells, $Ca^{2+}$ blocking agents could be a solution. Photofrin delivery to intrahepatic tumours might be improved by increasing tumour capillary flow with appropriate vasoactive agents (Ackerman *et al.*, 1988). The mechanism responsible for porphyrin accumulation in tumours is still unclear. In a recent study, however, we showed that a decreased conversion to haem, owing to decreased ferrochelatase activity may be an important factor (Van Hillegersberg *et al.*, 1992*b*). Dailey & Smith (1984) have shown that several representative porphyrins in Photofrin are substrates for ferrochelatase. Therefore, modulation of this enzyme could be another approach (Smith, 1987).

As the diffuser was inserted into the centre of the tumour, higher light doses were applied to the tumour than to the surrounding liver tissue. However, on the second day after treatment at all energies a rim of liver necrosis was visible around the tumour, whereas at lower energies tumour tissue had remained largely intact. This could be the result of the unfavourable porphyrin concentration ratio between liver and tumour, which was found to be 3:1 after i.v. Photofrin in this model (Van Hillegersberg *et al.*, 1992*b*).

In this study liver necrosis increased with energy delivered, but a plateau occurred beyond 400 J $cm^{-1}$, indicating that higher laser energies could be applied without much more liver damage. An important factor in this matter is the difference in optical properties between the dark red coloured liver and the whitish tumour (Van Hillegersberg *et al.*, 1992*c*). The hepatic tissue effectively absorbs 625 nm light, which limits the light penetration in liver, and by that limits liver damage. This difference in absorption coefficient might be beneficial in the clinical application, where the peripheral interstitial diffuser would produce a comparable amount of liver damage.

Serum ASAT and ALAT levels on the first and second day after treatment were used as biochemical parameters for liver damage. Indeed a significant relation between enzyme level and delivered light energy was found, confirming our measurements on the area of hepatic necrosis in the experimental group. However, the rise in ASAT level after diffuser insertion only, did not correspond to the normal histological appearance of surrounding liver tissue. Recently we measured ASAT and ALAT concentrations in rat liver and tumour CC531. The ALAT concentration ratio between tumour and liver was found to be 1:40, whereas ASAT ratio was 1:2, indicating that the serum ALAT level is the most appropriate parameter for liver damage in this model (unpublished data). Thus the increase in serum ASAT after diffuser insertion is probably due to tumour, rather than liver damage. A similar explanation could be applied to the rise in ASAT level after treatment with light only, especially at energies $\leqslant$ 400 J $cm^{-1}$. In this group liver necrosis appeared at 800 and 1600 J $cm^{-1}$, resulting in a higher ALAT level (Figures 7 and 8).

In this study we used a single PDT treatment with a fixed diffuser position to determine the photodynamic effects as standardised as possible. We therefore chose a tumour model in the rat, where the mean diameter of the tumour at time of treatment was approximately 6 mm so that the effects on the tumour and on the adjacent liver tissue could be determined. Larger tumours, however, could be treated by multiple diffuser implantation (Marijnissen *et al.*, 1992). In this respect, fluence measurements at the tumour boundary would give important information to compare with the light dose delivered in this study (i.e. $170 \pm 30$ J $cm^{-2}$ with 400 J $cm^{-1}$ from the diffuser). As the diffuser can be passed down a needle under ultrasound guiding, percutaneous treatment of several tumours at different liver lobes would be possible (Gatenby *et al.*, 1987). This local treatment of tumour can substantially reduce the surgical trauma and may as such diminish complications like bleeding and liver failure.

This study was supported by a grant from The Netherlands Digestive Disease Foundation (WS 89-14). The authors wish to thank Mrs O. Pelgrim for her work on histology, Mr F. van der Panne for photography, Mrs L. Sorber for programming the IBAS, Mr W.P. van Scalkwijk for his advice and for carrying out the biochemical determinations and Drs P.G. Mulder for his advice concerning statistical evaluation.

### References

ACKERMAN, N.B., JACOBS, R., BLOOM, N.D. & POON, T.T. (1988). Increased capillary flow in intrahepatic tumours due to α-adrenergic effects of catecholamines. *Cancer*, **61**, 1550-1554.

ARNFIELD, M.R., TULIP, J., CHETNER, M. & MCPHEE, M.S. (1989). Optical dosimetry for interstitial photodynamic therapy. *Med. Phys.*, **16**, 602-608.

ARNFIELD, M., GONZALEZ, S., LEA, P., TULIP, J. & MCPHEE, M. (1986). Cylindrical irradiation fibre tip for photodynamic therapy. *Lasers Surg Med.*, **6**, 150-154.

BLACHUM, O.J. & DOIRON, D.R. (1985). Photoradiation therapy of endobronchial lung cancer: large obstructing tumours, non-obstructing tumours and early stage bronchial cancer lesions. *Clin. Chest. Med.*, **6**, 255-275.

BELLNIER, D.A., HO, Y.K., PANDEY, R.K., MISSERT, J.R. & DOUGHERTY, T.J. (1989). Distribution and elimination of Photofrin II in mice. *Photochem. Photobiol.*, **50**, 221-228.

BONNET, R. & BERENBAUM, M. (1989). Phorphyrins as photosensitizers. *Ciba Found. Symp.*, **146**, 41-59.

BROWN, S.G., TRALAU, C.J., COLDERIDGE SMITH, P.D., AKDEMIR, D. & WIEMAN, T.J. (1986). Photodynamic therapy with porphyrin and phtalocyanine sensitisation: quantitative studies in normal rat liver. *Br. J. Cancer*, **54**, 45-52.

BUGELSKI, P.J., PORTER, C.W. & DOUGHERTY, T.J. (1981). Autoradiographic distribution of hematoporphyrin derivative in normal and tumour tissue of the mouse. *Cancer Res.*, **41**, 4606-4612.

BYCZKOWSKI, J.Z. & GESSNER, T. (1988). Biological role of superoxide ion-radical. *Int. J. Biochem.*, **20**, 569-580.

COZZANI, I., JORI, G., REDDI, E., TOMIO, L., ZORAT, P.L., SICURO, T. & MALVALDI, G. (1984). Interaction of free and liposome-bound porphyrins with normal and malignant cells: biochemical and photosensitization studies *in vitro* and *vivo*. In: Andreoni, A. & Cubbedu R. (eds) *Porphyrins in Tumour Therapy*, Plenum Press: New York and London, pp. 157-165.

DAILEY, H.A. & SMITH, A. (1984). Differential interaction of porphyrins used in photoradiation therapy with ferrochelatase. *Biochem. J.*, **223**, 441-445.

DOIRON, D.R., SVAASAND, L.O. & PROFIO, A.E. (1983). Light dosimetry and tissue: application in photoradiation therapy. *Adv. Exp. Med. Biol.*, **160**, 63-76.

DOUGHERTY, T.J. (1987). Photosensitizers: therapy and detection of malignant tumours. *Photochem. Photobiol.*, **45**, 879-889.

DOUGHERTY, T.J. (1989). Photodynamic therapy – new approaches. *Semin. Surg. Oncol.*, **5**, 6-16.

DOUGHERTY, T.J., HOMA, R.E., BOYLE, D.G. & WEISHAUPT, K.R. (1981). Interstitial photoradiation therapy for primary solid tumours in pet cats and dogs. *Cancer Res.*, **41**, 401-404.

DOUGHERTY, T.J., POTTER, W.R. & WEISHAUPT, K.R. (1984). The structure of the active component of hematoporphyrin derivative. In *Porphyrin Localization and Treatment of Tumours*. Doiron, D.R. & Gomer, C.J. (eds), Alan R. Liss Inc.: New York, pp. 301-314.

FINGAR, V.H., WIEMAN, T.J. & DOAK, K.W. (1990). Role of thromboxane and prostacyclin release on photodynamic therapy-induced tumour destruction. *Cancer Res.*, **50**, 2599-2603.

FOOTE, C.S. (1991). Definition of type I and type II photosensitized oxidation. *Photochem. Photobiol.*, **54**, 659.

GATENBY, R.A., NANCY, D. & BROWN, D.Q. (1987). Tumour therapy with hematoporphyrin derivative and laser via a percutaneous fiberoptic technique: preclinical experiments. *Radiology*, **163**, 167–171.

GOMER, C.J. & DOUGHERTY, T.J. (1979). Determination of (3H)- and (14C) hematoporphyrin derivative distribution in malignant and normal tissue. *Cancer Res.*, **39**, 146–151.

GOMER, C.J. (1989). Photodynamic therapy in the treatment of malignancies. *Semin Hematol.*, **26**, 27–34.

GOMER, C.J., RUCKER, R.N., FERRARIO, A. & WONG, S. (1989). Properties and applications of photodynamic therapy. *Radiat Res.*, **120**, 1–18.

GUTTERIDGE, J.M.C. & HALLIWELL, B. (1990). The measurement and mechanism of lipid peroxidation in biological systems. *TIBS*, **15**, 129–135.

HENDERSON, B.W. & BELLNIER, D.A. (1989). Tissue localization of photosensitizers and the mechanism of photodynamic tissue destruction. *Ciba Found. Symp.*, **146**, 113–130.

HOLT, S., TULIP, J., HAMILTON, D., CUMMINS, J., FIELDS, A. & DICK, C. (1985). Experimental laser phototherapy of the morris 7777 hepatoma in the rat. *Hepatology*, **5**, 175–180.

JORI, G., REDDI, E., COZZANI, I. & TOMIO, L. (1986). Controlled targeting of different subcellular sites by porphyrins in tumour-bearing mice. *Br. J. Cancer*, **53**, 615–621.

JUNGERMANN, K. & KATZ, N. (1989). Functional specialization of different hepatocyte populations. *Phys. Rev.*, **69**, 709–764.

KEENE, J.P., KESSEL, D., LAND, E.J., REDMOND, R.W. & TRUSCOTT, T.G. (1986). Direct detection of singlet oxygen sensitized by hematoporphyrin and related compounds. *Photochem. Photobiol.*, **43**, 117–120.

KESSEL, D., THOMPSON, P., MUSSELMAN, B. & CHANG, C.K. (1987). Probing the structure and stability of the tumour localizing derivative of hematoporphyrin by reduction with LiA1H4. *Cancer Res.*, **47**, 4642–4645.

KITA, K., ITAOSHIMA, T., ITO, T. & 12 others (1987). Photodynamic therapy of rat liver cancer: protection of the normal liver by indocyanine green. *Gastroen. Japon.*, **22**, 465–473.

LAMERS, H.L., MOORMAN, F.M. & CHARLES, R. (1989). The metabolic lobulus, a key to the architecture of the liver. *RBC*, **19**, 5–26.

LIPSON, R.L., BALDES, E.J. & OLSEN, A.M. (1961). The use of hematoporphyrin in tumour destruction. *J. Natl Cancer Inst.*, **26**, 1–11.

MANYAK, M.J., RUSSO, A., SMITH, P.D. & GLATSTEIN, E. (1988). Photodynamic therapy. *J. Clin. Oncol.*, **6**, 380–391.

MARIJNISSEN, J.P.A. & STAR, W.M. (1987). Quantitative light dosimetry *in vitro* and *in vivo*. *Lasers Med. Sci.*, **2**, 235–241.

MARIJNISSEN, J.P.A., STAR, W.M., VERSTEEG, A.A.C. & FRANKEN, N.A.P. (1985). Pilot study on the interstitial HPD-PDT in rats bearing solid mammary carcinoma or rhabdomyosarcoma. In *Photodynamic Therapy of Tumours and Other Diseases*. Jori, G. & Perria, C. (eds). Liberia Progetto: Padova, pp. 387–390.

MARIJNISSEN, J.P.A., VERSTEEG, J.A.C., STAR, W.M. & VAN PUTTEN, W.L.J. (1992). Tumour and normal tissue response to interstitial photodynamic therapy of the rat R-1 rhabdomyosarcoma. *Int. J. Radiat. Oncol. Biol. Phys.*, **22**, 936–972.

MARQUET, R.L., WESTBROEK, D.L. & JEEKEL, J. (1984). Interferon treatment of a transplantable colon carcinoma: importance of tumour site. *Int. J. Cancer*, **33**, 689–692.

MATSUMOTO, N., SAITO, N., MIYOSHI, N., NAKANISHI, K. & FUKUDA, M. (1990). Combination effect of hyperthermia and photodynamic therapy on colon carcinoma. *Head Neck Surg.*, **116**, 824–829.

NISHIWAKI, Y., NAKAMURA, S. & SAKAGUCHI, S. (1989). New method of photosensitizer accumulation for photodynamic therapy in an experimental liver tumour. *Lasers Surg. Med.*, **9**, 254–263.

PIMSTONE, N.R., HORNER, I.J., SHAYLOR-BILLINGS, J. & GANDHI, S.N. (1982). Hematoporphyrin-augmented phototherapy in experimental liver cancer in the rat. *Soc. Photo-Opt. Instrum. Eng.*, **357**, 60–67.

SHARGEL, L., CHEUNG, W.M. & YU, A.B.C. (1979). High-pressure liquid chromatographic analysis of antipyrine in small plasma samples. *J. Pharm. Sci.*, **68**, 1052–1054.

SMITH, A. (1987). Mechanisms of toxicity of photoactivated artificial porphyrins: role of phorphyrin–protein interaction. *Ann. N.Y. Acad. Sci.*, **514**, 309–322.

STAR, W.M., MARIJNISSEN, J.P.A., VAN DEN BERG-BLOK, A.E., VERSTEEG, J.A.C., FRANKEN, K.A.P. & REINHOLD, H.S. (1986). Destruction of rat mammary tumour and normal tissue microcirculation by hematoporphyrin derivative photoradiation observed in vivo in sandwich observation chambers. *Cancer Res.*, **46**, 2532–2540.

STAR, W.M., VERSTEEG, A.A.C., VAN PUTTEN, W. & MARIJNISSEN, J.P.A. (1990). Wavelength dependence of hematoporphyrin derivative photodynamic treatment on rat ears. *Photochem. Photobiol.*, **52**, 547–554.

STEELE, G. & RAVIKUMAR, T.S. (1989). Resection of hepatic metastases from cancer: biologic prospectives. *Ann. Surg.*, **210**, 127–138.

SUGARBAKER, P.H. & KEMENY, N. (1989). Management of metastatic cancer to the liver. *Adv. Surg.*, **22**, 1–56.

VAN HILLEGERSBERG, R., KORT, W.J., TEN KATE, F.J.W. & TERPSTRA, O.T. (1991). Water-jet-cooled Nd:YAG laser coagulation: selective destruction of rat liver metastases. *Lasers Surg. Med.*, **11**, 445–454.

VAN HILLEGERSBERG, R., KORT, W.J., VERMEIJ, M. & TERPSTRA, O.T. (1992*a*). Treatment of experimental liver metastases with a noncontact Neodymium: YAG laser. *J. Surg. Res.*, **53**, 128–135.

VAN HILLEGERSBERG, R., VAN DEN BERG, J.W.O., KORT, W.J., TERPSTRA, O.T. & WILSON, J.H.P. (1992*b*). Selective accumulation of endogenously produced prophyrins in a liver metastases model in rats. *Gastroenterology*, **103**, 647–651.

VAN HILLEGERSBERG, R., PICKERING, J.W., AALDERS, M. & BEEK, F.B. (1992*c*). Optical properties of rat liver and tumour at 633 nm and 1064 nm: photofrin enhances scattering. *Lasers Surg. Med.* (in press).

WAGMAN, L.D., KEMENY, M.M., LEONG, L. & 5 others (1990). A prospective, randomized evaluation of the treatment of colorectal cancer metastatic to the liver. *J. Clin. Oncol.*, **8**, 1885–1893.

WALDOW, S.M. & DOUGHERTY, T.J. (1984). Interaction of hyperthermia and photoradiation therapy. *Radiat. Res.*, **97**, 380–385.

WALDOW, S.M., HENDERSON, B.W. & DOUGHERTY, T.J. (1987). Hyperthermic potentiation of photodynamic therapy employing Photofrin I and II: comparison of results using three animal tumour models. *Lasers Surg. Med.*, **7**, 12–22.

WEISHAUPT, K.R., GOMER, C.J. & DOUGHERTY, T.J. (1976). Identification of singlet oxygen as the cytotoxic agent in photoactivation of a murine tumour. *Cancer Res.*, **36**, 2326–2329.

WILSON, J.H.P., VAN HILLEGERSBERG, R., VAN DEN BERG, J.W.O., KORT, W.J. & TERPSTRA, O.T. (1991). Photodynamic therapy for gastrointestinal tumours. *Scand. J. Gastroenterol.*, **26** Suppl. 188, 11.